# Western Kentucky Dental Association

**Published:** 1897-03

**Authors:** W. H. Pitcher

**Affiliations:** Paducah, Ky.


*Editor of the Register:*

DEAR SIR: The dentists of south-western Kentucky met in the council chamber of the City Hall in Paducah, Ky., Friday, January 15, 1897, and formed a dental association to be known as the Western Kentucky Dental Association, and elected the following officers for the ensuing year: President, Dr. W. H. Pitcher, Paducah, Ky.; vice-president, Dr. R. B. Collie, Birmingham, Ky.; secretary, Dr. W. L. Hansbro, Paducah, Ky.; treasurer, Dr. J. Reynolds, Mayfield, Ky. The next meeting will be held in Paducah, Ky., April 20, 1897.

Yours truly,

W. H. PITCHER.

PADUCAH, KY., Jan. 16, 1897.

---

DURING 1896 Chicago did not have a single case of smallpox.

---

ARRANGEMENTS are being perfected for the forthcoming semi-centennial meeting of the American Medical Association in the new Horticultural Hall, of Philadelphia.

